# Supplementary material for: Implementation Strategies Used to Increase Human Papillomavirus Vaccination Uptake by Adolescent Girls in Sub-Saharan Africa: A Scoping Review
Source: Vaccines (Basel). 2023 Jul 16;11(7):1246. doi: 10.3390/vaccines11071246 (PMC10385137; doi:10.3390/vaccines11071246)
Supplement: Supplementary file 1 [file vaccines-11-01246-s001.zip › vaccines-2426520-supplementary.pdf]

Table S1. PubMed search strategy. PubMed Search 26.01.2022.

Search	Actions	Details	Query	Results	Time
#6			Search: **((((#1) AND (#2)) AND (#3)) AND (#4)) AND (#5)**	142	07:38:37
#5			Search: **(((sub-Saharan Africa) OR (Africa south Sahara)) OR (SSA)) OR (Angola)) OR (Benin)) OR (Burkina Faso)) OR (Botswana)) OR (Burundi)) OR (Cameroon)) OR (Cape Verde)) OR (Central Africa Republic)) OR (Chad)) OR (Congo)) OR (Cote d'voire)) OR (Djibouti)) OR (Eritrea)) OR (Ethiopia)) OR (The Gambia)) OR (Ghana)) OR (Guinea)) OR (Guinea-Bissau)) OR (Gabon)) OR (Kenya)) OR (Lesotho)) OR (Liberia)) OR (Madagascar)) OR (Malawi)) OR (Mali)) OR (Mauritania)) OR (Mauritius)) OR (Mozambique)) OR (Namibia)) OR (Niger)) OR (Nigeria)) OR (Rwanda)) OR (Sao tome principle)) OR (Senegal)) OR (seychelles)) OR (sierra Leon)) OR (Somalia)) OR (South Africa)) OR (Sudan)) OR (south Sudan)) OR (Swaziland)) OR (United republic of Tanzania)) OR (Togo)) OR (Uganda)) OR (Zimbabwe)) OR (Zambia)**	635,885	07:37:13
#4			Search: **(Uptake) OR (coverage)**	565,814	07:35:27
#3			Search: **((Human papillomavirus vaccination) OR (HPV vaccination)) OR (HPV immunization)**	18,234	07:34:36
#2			Search: **(Implementation strategies) OR (interventions)**	9,425,631	07:33:47
#1			Search: **((((((Girls) OR (parents)) OR (Teachers)) OR (Health care providers)) OR (health professionals)) OR (doctors)) OR (nurses)**	3,168,662	07:31:21

Table S2. PRISMA-ScR checklist. Preferred Reporting Items for Systematic Reviews and Meta-Analyses extension for Scoping Reviews (PRISMA-ScR) Checklist.

SECTION	ITEM	PRISMA-ScR CHECKLIST ITEM	REPORTED ON PAGE #
		**TITLE**	
Title	1	Implementation strategies used to increase human papillomavirus vaccination uptake by adolescent girls in sub-Saharan Africa: A scoping review	1
		**ABSTRACT**	
Structured summary	2	Abstract provided	1
		**INTRODUCTION**	
Rationale	3	This review provides a basis for future research on increasing the uptake of the HPV vaccine by adolescent girls as we reported importance and feasibility of interventions.	4
Objectives	4	To identify implementation strategies used to increase HPV vaccination uptake by adolescent girls in sub-Saharan Africa, report their importance and feasibility using the Expert recommendation for implementation change compilation, and form a basis for future systematic reviews to evaluate effectiveness.	4
		**METHODS**	
Protocol and registration	5	Protocol was published: Lubeya MK, Mwanahamuntu M, Chibwesha C, Mukosha M, Wamunyima MM, Kawonga M. Implementation strategies to increase human papillomavirus vaccination uptake for adolescent girls in sub-Saharan Africa: A scoping review protocol. PloS one.	5

		2022;17(8):e0267617. Epub 2022/08/26. doi: 10.1371/journal.pone.0267617.	
Eligibility criteria	6	We captured all research papers published in English on implementation strategies including various stakeholders (parents, adolescent girls, teachers, and community leaders between January 2006 and December 2021.	5
Information sources*	7	The databases searched included: PubMed, EMBASE, CINAHL (Cumulative Index to Nursing and Allied Health Literature) (EBSCO), Google Scholar, and Scopus. Gray literature citations were also searched including Google Scholar.	5
Search	8	Provided as S1	S1 Appendix 1
Selection of sources of evidence†	9	Two independent reviewers assessed articles for inclusion by screening titles, abstracts, and full text.	7
Data charting process‡	10	The data extracted include details about the participants, concept, context, study methods, and key findings relevant to the review question. The draft charting tool was piloted on five included articles, and revisions were made as necessary during data charting.	7
Data items	11	Table 1	8
Critical appraisal of individual sources of evidence§	12	NA	NA
Synthesis of results	13	The extracted data have been presented in Tables and supplementary files to align with the study objectives, accompanied by a narrative summary of how the findings relate to the research question and objectives.	8
		**RESULTS**	
Selection of sources of evidence	14		Click here to enter text.
Characteristics of sources of evidence	15		Click here to enter text.
Critical appraisal within sources of evidence	16		Click here to enter text.
Results of individual sources of evidence	17		Click here to enter text.
Synthesis of results	18		Click here to enter text.
		**DISCUSSION**	
Summary of evidence	19	We identified and mapped implementation strategies to increase HPV vaccination uptake for adolescent girls in sub-Saharan Africa according to ERIC compilation and reported their importance and feasibility.	18
Limitations	20	This scoping review only focused on studies carried out in English and may have missed other important research in other languages. Furthermore, there are limitations inherent to the scoping review methodology itself.	20
Conclusions	21		21
		**FUNDING**	
Funding	22	MKL and MM are supported by the UNC-UNZA-Wits Partnership for HIV and Women's Reproductive Health Grant number: D43 TW010558.	22

JBI = Joanna Briggs Institute; PRISMA-ScR = Preferred Reporting Items for Systematic Reviews and Meta-Analyses extension for Scoping Reviews.

* Where *sources of evidence* (see second footnote) are compiled from, such as bibliographic databases, social media platforms, and Web sites.

† A more inclusive/heterogeneous term used to account for the different types of evidence or data sources (e.g., quantitative and/or qualitative research, expert opinion, and policy documents) that may be eligible in a scoping review instead of only studies. This is not to be confused with *information sources* (see first footnote).

‡ The frameworks by Arksey and O'Malley (6) and Levac et al. (7) and the JBI guidance (4, 5) refer to the process of data extraction in a scoping review as data charting.

§ The process of systematically examining research evidence to assess its validity, results, and relevance before using it to inform a decision. This term is used for items 12 and 19 instead of "risk of bias" (which is more applicable to systematic reviews of

interventions) to include and acknowledge the various sources of evidence that may be used in a scoping review (e.g., quantitative and/or qualitative research, expert opinion, and policy document).

From: Tricco AC, Lillie E, Zarin W, O'Brien KK, Colquhoun H, Levac D et al. PRISMA Extension for Scoping Reviews (PRISMA-ScR): Checklist and Explanation. Ann Intern Med. 2018;169:467–473. doi: 10.7326/M18-0850.

Table S3. Identified strategies per study according to ERIC compilation.

SN	Implementation strategy (ERIC)	Definition	Binagwaho, Agnes (2012)	LaMontagne, D Scott (2011)	Watson, Deborah (2012)	Casey, Rabecca M (2021)	Galagan, Sean R (2013)	Raesima, Mmakgomo M (2015)	Gallagher, Katherine E (2017)	Ladner, Joel (2012)	Chigbu, Chibuike O (2017)	World Health Organization (2015)	Kabakama, Severin (2016)	Moodley, I (2013)	Wamai, Richard (2012)	Msyamboza, Kelias P (2017)	Mugisha, Emmanuel (2015)	Tsu, Vivien (2021)	Snyman, Leon (2015)	Whirtworth, Hilary (2021)	Drokow, Emmanuel K (2021)	Mpuru, Alex (2021)	Ladner, Joe (2016)	Soi, Catherine (2018)	Delany-Muretwe (2018)	LaMontagne, Scott D (2021)	Engel, Danielle (2021)	Jones, Amy (2021)	PATH, (2011)	Ministry of Health, Zambia (2013)	Total times strategy used
1	Access new funding	Access new or existing money to facilitate the implementation	1	1	1	1	0	1	1	1	0	1	0	1	0	1	0	1	1	1	0	1	1	1	1	1	1	0	1	1	**21**
2	Alter incentive/allowance structures	Work to incentivize the adoption and implementation of the clinical innovation	0	0	0	0	0	0	0	0	0	0	0	0	0	0	0	0	0	0	0	0	0	0	0	0	0	0	0	0	**0**
3	Alter patient/consumer fees	Create fee structures where patients/consumers pay less for preferred treatments (the clinical innovation) and more for less-preferred treatments	0	0	0	0	0	0	0	0	0	0	0	0	0	0	0	0	0	0	0	0	0	0	0	0	0	0	0	0	**0**

4	Assess for readiness and identify barriers and facilitators	Assess various aspects of an organization to determine its degree of readiness to implement barriers that may impede implementation, and strengths that can be used in the implementation effort	1	1	1	1	0	1	0	1	0	0	1	1	1	0	0	1	0	1	0	1	1	1	1	1	1	0	1	1	**19**
5	Audit and provide feedback	Collect and summarize clinical performance data over a specified time period and give it to clinicians and administrators to monitor, evaluate, and modify provider behavior	0	0	0	1	0	1	1	1	0	0	1	0	0	0	0	0	0	0	0	1	1	0	1	1	1	0	1	1	**12**
6	Build a coalition	Recruit and cultivate relationships with partners in the implementation effort	1	1	1	1	0	1	1	1	0	1	1	1	1	1	1	1	1	1	0	1	1	1	1	1	1	0	1	1	**24**

7	Capture and share local knowledge	Capture local knowledge from implementation sites on how implementers and clinicians made something work in their setting, and then share it with other sites	0	0	0	1	0	1	0	1	0	0	1	1	0	0	1	1	1	0	0	1	1	0	0	0	1	0	1	0	**12**
8	Centralize technical assistance	Develop and use a centralized system to deliver technical assistance focused on implementation issues	1	0	1	1	0	1	1	0	0	0	0	1	0	1	1	1	0	1	0	1	1	1	0	1	1	0	1	1	**17**

9	Change accreditation or membership requirements	Strive to alter accreditation standards in order that they require or encourage use of the clinical innovation. Work to alter membership organization requirements in order that those who want to affiliate with the organization are encouraged or required to use the clinical innovation	0	0	0	0	0	0	0	0	0	0	0	0	0	0	0	0	0	0	0	0	0	0	0	0	0	0	0	0	**0**
10	Change liability laws	Participate in liability reform efforts that make clinicians more willing to deliver the clinical innovation	0	0	0	0	0	0	0	0	0	0	0	0	0	0	0	0	0	0	0	0	0	0	0	0	0	0	0	0	**0**

11	Change physical structure and equipment	Evaluate current configurations and adapt, as needed, the physical structure and/or equipment (*e.g.*, changing the layout of a room, adding equipment) to best accommodate the targeted innovation	0	1	0	0	0	0	0	0	0	0	0	1	0	0	0	0	0	0	0	0	1	0	0	0	0	0	1	1	**5**
12	Change record systems	Change record systems to allow for better assessment of implementation or clinical outcomes	0	0	0	0	0	0	0	0	0	0	0	0	0	0	0	0	0	0	0	0	1	0	1	0	0	0	0	0	**2**
13	Change service sites	Change the location of clinical service sites to increase access	1	1	1	1	0	1	1	1	0	1	1	1	1	1	1	1	1	1	0	1	1	1	1	1	1	0	1	1	**24**

14	Conduct cyclical small tests of change	Implement changes in a cyclical fashion using small tests of change before taking changes system-wide. Tests of change benefit from systematic measurement, and results of the tests of change are studied for insights on how to perform better. This process continues serially over time, and refinement is added with each cycle	0	0	0	0	0	0	0	0	0	0	1	0	0	0	1	1	1	1	0	1	1	0	0	0	0	0	0	1	**8**

15	Conduct educational meetings	Hold meetings targeted toward different stakeholder groups (*e.g.*, providers, administrators, other organizational stakeholders and community, patient/consumer and family stakeholders) to teach them about the clinical innovation	1	1	1	1	1	1	1	1	1	0	1	1	0	0	0	1	1	1	1	1	1	1	1	1	1	0	1	1	**23**
16	Conduct educational outreach visits	Have a trained person meet with providers in their practice settings to educate providers about the clinical innovation with the intent of changing the provider's practice	0	0	0	1	0	1	0	1	1	0	0	1	1	0	1	1	0	1	0	1	1	1	1	1	1	0	1	0	**16**

17	Conduct local consensus discussions	Include local providers and other stakeholders in discussions that address whether the chosen problem is important and whether the clinical innovation to address it is appropriate	1	0	1	1	1	1	0	1	1	0	1	1	0	0	0	1	1	1	0	1	1	1	1	1	1	0	1	1	20
18	Conduct local needs assessment	Collect and analyze data related to the need for the innovation	1	0	0	1	0	1	0	0	1	0	0	0	0	0	1	1	0	0	0	1	0	1	1	1	0	0	1	1	12
19	Conduct ongoing training	Plan for and conduct training in the clinical innovation in an ongoing way	0	0	1	1	0	0	0	0	1	0	0	0	0	0	1	1	0	0	0	1	1	0	0	1	1	0	1	1	11

20	Create a learning collaborative environment	Facilitate the formation of groups of providers or provider organizations and foster a collaborative learning environment to improve implementation of the clinical innovation	1	0	0	1	0	1	0	0	1	0	0	1	1	0	0	1	0	1	0	1	1	0	0	0	0	0	1	0	11
21	Create new clinical teams	Change who serves on the clinical team, adding different disciplines and different skills to make it more likely that the clinical innovation is delivered (or is more successfully delivered)	0	0	0	0	0	0	0	0	0	0	0	1	0	0	0	0	0	0	0	0	0	0	0	0	0	0	0	0	1

22	Create or change credentialing and/or licensure standards	Create an organization that certifies clinicians in the innovation or encourage an existing organization to carry this out. Change governmental professional certification or licensure requirements to include delivering the innovation. Work to alter continuing education requirements to shape professional practice toward the innovation	0	0	0	0	0	0	0	0	0	0	0	0	0	0	0	0	0	0	0	0	0	0	0	0	0	0	0	0	0

23	Develop a formal implementation blueprint	Develop a formal implementation blueprint that includes all goals and strategies. The blueprint should include the following: 1) aim/purpose of the implementation; 2) scope of the change (*e.g.*, what organizational units are affected); 3) timeframe and milestones; and 4) appropriate performance/progress measures. Use and update this plan to guide the implementation effort over time	1	0	0	1	0	1	0	1	1	0	0	1	0	0	0	1	1	0	0	1	0	1	1	1	0	0	1	0	13

24	Develop academic partnerships	Partner with a university or academic unit for the purposes of shared training and bringing research skills to an implementation project	0	0	0	0	0	0	0	0	0	0	0	0	0	0	0	0	0	1	0	1	0	0	0	0	0	1	0	0	3
25	Develop an implementation glossary	Develop and distribute a list of terms describing the innovation, implementation, and stakeholders in the organizational change	0	0	0	1	0	0	0	0	0	0	0	0	0	0	0	0	0	0	0	1	0	0	0	0	0	0	0	0	2

26	Develop and implement tools for quality monitoring	Develop, test, and introduce into quality-monitoring systems the right input—the appropriate language, protocols, algorithms, standards, and measures (of processes, patient/consumer outcomes, and implementation outcomes) that are often specific to the innovation being implemented	0	0	0	1	0	0	0	0	0	0	0	0	0	0	0	0	0	0	0	0	1	0	0	0	0	0	1	0	3
27	Develop and organize quality monitoring systems	Develop and organize systems and procedures that monitor clinical processes and/or outcomes for the purpose of quality assurance and improvement	0	0	0	1	0	0	0	0	0	0	0	1	1	0	0	0	0	1	0	1	1	0	1	0	0	0	1	1	9

28	Develop disincentives	Provide financial disincentives for failure to implement or use the clinical innovations	0	0	0	0	0	0	0	0	0	0	0	0	0	0	0	0	0	0	0	0	0	0	0	0	0	0	0	0	0
29	Develop educational materials	Develop and format manuals, toolkits, and other supporting materials in ways that make it easier for stakeholders to learn about the innovation and for clinicians to learn how to deliver the clinical innovation	1	1	1	1	;	1	1	1	1	0	1	1	1	0	1	1	1	0	0	1	1	1	1	1	1	1	1	0	22
30	Develop resource sharing agreements	Develop partnerships with organizations that have resources needed to implement the innovation	1	1	0	1	0	1	1	1	0	1	1	0	1	1	0	1	1	0	0	1	1	0	1	1	1	0	1	0	18

31	Distribute educational materials	Distribute educational materials (including guidelines, manuals, and toolkits) in person, by mail, and/or electronically	1	1	1	1	1	1	1	1	1	0	1	1	1	0	1	1	1	0	1	1	1	1	1	1	1	0	1	0	23
32	Facilitate relay of clinical data to providers	Provide as close to real-time data as possible about key measures of process/outcomes using integrated modes/channels of communication in a way that promotes use of the targeted innovation	0	0	0	1	0	1	0	0	0	0	0	0	0	0	0	1	0	0	0	0	0	0	0	0	0	0	1	1	5
33	Facilitation	A process of interactive problem solving and support that occurs in a context of a recognized need for improvement and a supportive interpersonal relationship	0	0	1	0	1	0	0	0	0	0	1	1	1	0	0	0	0	0	0	1	1	1	0	0	1	0	1	1	11

34	Fund and contract for the clinical innovation	Governments and other payers of services issue requests for proposals to deliver the innovation, use contracting processes to motivate providers to deliver the clinical innovation, and develop new funding formulas that make it more likely that providers will deliver the innovation	0	0	0	0	0	0	0	0	0	0	0	0	0	0	0	0	0	0	0	0	0	0	0	0	0	0	0	0	0

35	Identify and prepare champions	Identify and prepare individuals who dedicate themselves to supporting, marketing, and driving through an implementation, overcoming indifference or resistance that the intervention may provoke in an organization	1	0	1	1	1	0	0	0	0	1	1	0	0	0	0	0	0	0	0	1	0	1	0	0	0	0	1	0	9
36	Identify early adopters	Identify early adopters at the local site to learn from their experiences with the practice innovation	0	0	0	1	0	0	0	0	0	0	0	0	0	0	0	0	0	0	0	0	0	1	0	0	0	0	0	0	2
37	Increase demand	Attempt to influence the market for the clinical innovation to increase competition intensity and to increase the maturity of the market for the clinical innovation	0	0	0	0	0	0	0	0	0	0	0	0	0	0	0	0	0	1	0	1	1	0	0	0	0	0	0	0	3

38	Inform local opinion leaders	Inform providers identified by colleagues as opinion leaders or "educationally influential" about the clinical innovation in the hopes that they will influence colleagues to adopt it	1	0	1	1	1	0	1	0	0	0	0	0	0	1	0	0	0	0	0	1	1	1	1	0	0	0	1	1	12
39	Intervene with patients/consumers to enhance uptake and adherence	Develop strategies with patients to encourage and problem solve around adherence	0	0	0	0	1	0	0	1	0	0	1	1	0	1	0	1	0	1	0	1	1	1	1	1	1	0	0	0	13
40	Involve executive boards	Involve existing governing structures (*e.g.*, boards of directors, medical staff boards of governance) in the implementation effort, including the review of data on implementation processes	0	0	0	1	0	1	1	1	0	1	0	1	0	1	0	1	0	0	0	0	1	1	1	1	1	0	1	0	14

41	Involve patients/consumers and family members	Engage or include patients/consumers and families in the implementation effort	1	1	1	1	1	1	0	1	0	0	1	1	1	1	1	1	0	1	0	1	1	1	1	1	1	1	1	0	22
42	Make billing easier	Make it easier to bill for the clinical innovation	0	0	0	0	0	0	0	0	0	0	0	0	0	1	1	0	0	0	0	0	0	0	0	0	0	0	0	0	2
43	Make training dynamic	Vary the information delivery methods to cater to different learning styles and work contexts, and shape the training in the innovation to be interactive	0	0	0	1	0	0	1	0	0	0	1	1	1	0	1	1	0	0	0	1	1	1	1	0	1	0	0	0	12
44	Mandate change	Have leadership declare the priority of the innovation and their determination to have it implemented	1	0	0	1	0	1	1	0	0	1	1	0	0	0	0	1	0	0	0	1	1	1	1	0	0	0	1	1	13
45	Model or simulate change	Model or simulate the change that will be implemented prior to implementation	0	0	0	0	0	0	0	0	0	0	0	0	0	0	0	0	0	0	0	0	0	0	0	0	0	0	0	0	0

46	Obtain and use patients/consumers and family feedback	Develop strategies to increase patient/consumer and family feedback on the implementation effort	0	0	0	0	0	1	0	0	0	0	0	0	1	0	0	0	0	0	0	0	1	1	0	1	1	0	1	0	7
47	Obtain formal commitments	Obtain written commitments from key partners that state what they will do to implement the innovation	1	1	0	1	0	0	0	1	0	0	0	0	0	0	0	1	0	0	0	1	1	1	0	1	1	0	1	1	12
48	Organize clinician implementation team meetings	Develop and support teams of clinicians who are implementing the innovation and give them protected time to reflect on the implementation effort, share lessons learned, and support one another’s learning	0	0	0	0	0	0	0	0	0	0	0	1	0	0	1	0	0	0	0	1	0	1	0	0	0	0	1	1	6

49	Place innovation on fee for service lists/formularies	Work to place the clinical innovation on lists of actions for which providers can be reimbursed (*e.g.*, a drug is placed on a formula, a procedure is now reimbursable)	0	0	0	0	0	0	0	0	0	0	0	0	0	0	0	0	0	0	0	0	0	0	0	0	0	0	0	0	0
50	Prepare patients/consumers to be active participants	Prepare patients/consumers to be active in their care, to ask questions, and specifically to inquire about care guidelines, the evidence behind clinical decisions, or about available evidence-supported treatments	0	0	0	0	0	0	0	0	0	0	0	0	0	0	0	1	0	1	0	1	1	0	0	1	0	0	0	0	5

51	Promote adaptability	Identify the ways a clinical innovation can be tailored to meet local needs and clarify which elements of the innovation must be maintained to preserve fidelity	1	1	0	1	1	1	1	1	0	0	1	1	0	1	1	1	1	0	0	1	1	1	1	1	1	0	1	1	21
52	Promote network weaving	Identify and build on existing high-quality working relationships and networks within and outside the organization, organizational units, teams, etc. to promote information sharing, collaborative problem-solving, and a shared vision/goal related to implementing the innovation	1	1	1	1	1	1	1	1	0	1	1	1	0	1	1	1	0	1	0	1	1	1	1	1	1	0	1	0	22

53	Provide clinical supervision	Provide clinicians with ongoing supervision focusing on the innovation. Provide training for clinical supervisors who will supervise clinicians who provide the innovation	0	0	0	0	1	1	1	1	0	0	0	0	1	0	0	0	1	0	0	1	1	0	0	1	1	0	1	1	12
54	Provide local technical assistance	Develop and use a system to deliver technical assistance focused on implementation issues using local personnel	0	0	0	0	1	1	0	1	0	0	1	0	1	0	0	0	0	0	0	1	1	0	1	0	1	0	1	1	11
55	Provide ongoing consultation	Provide ongoing consultation with one or more experts in the strategies used to support implementing the innovation	0	0	0	1	1	0	1	0	0	0	0	0	0	0	0	0	0	0	0	0	1	0	0	0	1	0	1	0	6

56	Purposely reexamine the implementation	Monitor progress and adjust clinical practices and implementation strategies to continuously improve the quality of care	0	0	0	1	1	1	0	0	0	0	0	0	0	0	0	0	0	0	0	1	1	0	1	0	0	0	1	1	8
57	Recruit, designate, and train for leadership	Recruit, designate, and train leaders for the change effort	0	0	0	1	0	0	1	0	0	0	0	1	0	1	0	0	0	0	0	0	0	0	0	0	0	0	0	0	4
58	Remind clinicians	Develop reminder systems designed to help clinicians to recall information and/or prompt them to use the clinical innovation	0	0	0	0	0	0	0	0	0	0	0	0	0	0	0	0	0	0	0	0	0	0	0	0	0	0	0	0	0
59	Revise professional roles	Shift and revise roles among professionals who provide care, and redesign job characteristics	0	0	0	0	0	0	0	0	0	0	0	1	0	0	0	0	0	0	0	0	0	0	0	0	0	0	1	0	2

60	Shadow other experts	Provide ways for key individuals to directly observe experienced people engage with or use the targeted practice change/innovation	0	0	0	0	0	0	0	0	0	0	0	1	0	0	0	0	0	0	0	0	0	0	0	0	0	0	0	0	1
61	Stage implementation scale up	Phase implementation efforts by starting with small pilots or demonstration projects and gradually move to a system wide rollout	1	1	1	1	1	1	1	1	0	1	1	1	0	1	1	1	1	0	0	1	0	1	1	1	1	0	1	1	22
62	Start a dissemination organization	Identify or start a separate organization that is responsible for disseminating the clinical innovation. It could be a for-profit or non-profit organization	0	0	0	1	0	0	0	0	0	0	0	0	0	0	0	0	0	0	0	0	0	0	0	0	0	1	0	0	2

63	Tailor strategies	Tailor the implementation strategies to address barriers and leverage facilitators that were identified through earlier data collection	0	0	0	1	1	1	1	1	1	0	1	1	0	1	1	1	0	1	0	1	1	1	1	1	1	1	1	1	21
64	Use advisory boards and workgroups	Create and engage a formal group of multiple kinds of stakeholders to provide input and advice on implementation efforts and to elicit recommendations for improvements	1	0	0	1	1	1	0	0	0	1	0	1	0	0	0	1	0	0	0	1	1	0	0	1	1	0	1	0	12
65	Use an implementation advisor	Seek guidance from experts in implementation	0	0	0	0	0	1	0	1	0	0	0	0	0	0	0	0	0	0	0	0	0	0	0	0	0	0	0	0	2
66	Use capitated payments	Pay providers or care systems a set amount per patient/consumer for delivering clinical care	0	0	0	0	0	0	1	0	0	0	0	0	0	0	1	0	0	0	0	0	0	1	0	0	0	0	0	0	3

67	Use data experts	Involve, hire, and/or consult experts to inform management on the use of data generated by implementation efforts	0	0	0	0	0	0	0	0	0	0	0	0	0	0	0	0	1	0	0	1	0	0	0	0	0	0	1	0	3
68	Use data warehousing techniques	Integrate clinical records across facilities and organizations to facilitate implementation across systems	0	0	0	0	0	0	0	0	0	0	0	0	0	0	0	0	0	0	0	0	0	0	0	0	0	0	0	0	0
69	Use mass media	Use media to reach large numbers of people to spread the word about the clinical innovation	1	0	1	0	1	1	1	1	1	0	1	1	1	0	1	1	0	1	0	1	1	1	1	1	1	1	1	1	22
70	Use other payment schemes	Introduce payment approaches (in a catch-all category)	0	0	0	0	0	0	0	0	1	0	0	0	0	0	0	0	0	0	0	0	0	0	0	0	0	0	0	0	1
71	Use train-the-trainer strategies	Train designated clinicians or organizations to train others in the clinical innovation	0	0	0	1	0	1	0	0	0	0	0	0	0	0	0	0	0	0	0	1	0	0	0	0	1	0	1	0	5

72	Visit other sites	Visit sites where a similar implementation effort has been considered successful	0	0	0	0	1	0	0	0	0	0	0	0	0	0	0	1	1	1	0	1	0	1	0	1	0	0	1	0	8
73	Work with educational institutions	Encourage educational institutions to train clinicians in the innovation	0	0	0	0	0	0	0	0	0	0	0	0	0	0	0	0	0	0	0	0	0	0	0	0	0	0	1	0	1
	Total number of strategies used per article		22	14	17	39	19	32	22	25	12	10	23	31	16	15	20	32	16	20	2	44	40	31	28	29	31	6	45	26	667

Table S4. Implementation strategies used ERIC clusters according to feasibility and importance.

Implementation strategies	No. of times identified	Go-Zone Quadrant Feasibility and Importance
1. Use evaluative and iterative strategies	113	
4 Assess for readiness and identify barriers and facilitators		
5 Audit and provide feedback	19	1
56 Purposefully re-examine the implementation	12	1
26 Develop and implement tools for quality moni-	8	1
toring	3	1
27 Develop and organize quality monitoring sys-	9	1
tems	13	1
23 Develop a formal implementation blueprint	12	1
18 Conduct local needs assessment	22	1
61 Stage implementation scale up	7	1
46 Obtain and use patients/consumers and family feedback	8	1
14 Conduct cyclical small tests of change		
2. Provide interactive assistance	51	
33 Facilitation	11	1
54 Provide local technical assistance	12	IV
53 Provide clinical supervision	11	IV
8 Centralize technical assistance	17	III
3. Adapt and tailor to context	45	
63 Tailor strategies	21	I
51 Promote adaptability	21	I
67 Use data experts	3	III
68 Use data warehousing techniques	0	III
4. Develop stakeholder interrelationships	164	
35 Identify and prepare champions	9	I
48 Organize clinician implementation team meetings	6	I
57 Recruit, designate, and train for leadership	4	IV
38 Inform local opinion leaders	12	I
6 Build a coalition	24	I
47 Obtain formal commitments	12	IV
36 Identify early adopters	2	I
17 Conduct local consensus discussions	20	I
7 Capture and share local knowledge	12	I
64 Use advisory boards and workgroups	12	I
65 Use an implementation advisor	2	I
45 Model and simulate change	0	II
72 Visit other sites	8	II
40 Involve executive boards	14	II
25 Develop an implementation glossary	2	II
24 Develop academic partnerships	3	II
52 Promote network weaving	22	III
5. Train and educate stakeholders	131	
19 Conduct ongoing training	11	I
55 Provide ongoing consultation	6	I

29 Develop educational materials	22	I
43 Make training dynamic	12	I
31 Distribute educational materials	23	I
71 Use train-the-trainer strategies	4	I
15 Conduct educational meetings	23	I
16 Conduct educational outreach visits	16	II
20 Create a learning collaborative environment	11	II
60 Shadow other experts	1	II
73 Work with educational institutions	1	II
6. Support clinicians	26	
32 Facilitate relay of clinical data to providers	5	I
58 Remind clinicians	0	II
30 Develop resource sharing agreements	18	III
59 Revise professional roles	2	III
21 Create new clinical teams	1	III
7. Engage consumers	65	
41 Involve patients/consumers and family members	22	I
39 Intervene with patients/consumers to enhance uptake and adherence	13	IV
50 Prepare patients/consumers to be active participants	5	IV
37 Increase demand	3	II
69 Use mass media	22	III
8. Utilize financial strategies	27	
34 Fund and contract for the clinical innovation	0	IV
1 Access new funding	21	IV
49 Place innovation on fee for service lists/formularies	0	IV
2 Alter incentive/allowance structures	0	III
42 Make billing easier	2	III
3 Alter patient/consumer fees	3	III
70 Use other payment schemes	1	III
28 Develop disincentives	0	III
66 Use capitated payments	0	III
9. Change infrastructure	46	
44 Mandate change	10	III
12 Change record systems	1	III
11 Change physical structure and equipment	5	III
22 Create or change credentialing and/or licensure standards	3	III
13 Change service sites	22	III
9 Change accreditation or membership requirements	0	III
62 Start a dissemination organization	2	III
10 Change liability laws	0	III

The rightmost column depicts the go-zone quadrant into which each strategy falls based on the scale mean cut-offs. Go-zone quadrant I: Importance and feasibility are both above the scale means. Go-zone quadrant II: Importance rating is lower and the feasibility rating is higher than the scale means. Go-zone quadrant III: Importance and feasibility ratings are both below scale means. Go-zone quadrant IV: Importance rating higher and feasibility lower than scale means according to Waltz et al.
